# Supplementary material for: The HUSH complex is a gatekeeper of type I interferon through epigenetic regulation of LINE-1s
Source: Nat Commun. 2020 Nov 3;11:5387. doi: 10.1038/s41467-020-19170-5 (PMC7609715; doi:10.1038/s41467-020-19170-5)
Supplement: Supplementary file 1 — Supplementary Information [file 41467_2020_19170_MOESM1_ESM.pdf]

Supplementary Information for:

The HUSH complex is a gatekeeper of type I interferon through epigenetic regulation of LINE-1s

Tunbak et al.

- Supplementary Figures 1-7
- Supplementary Tables 1-2

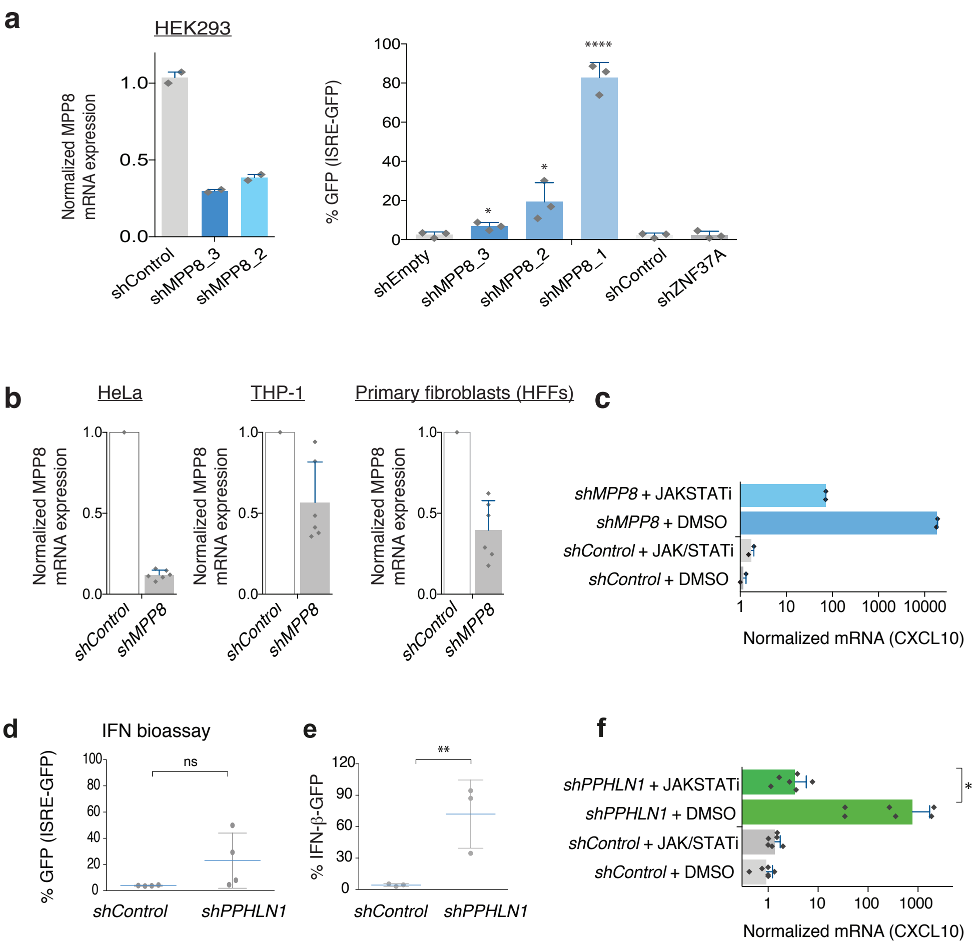

Supplementary Figure 1 MPP8 regulates the type I IFN response.
a Two additional shRNAs against MPP8 (shMPP8_2 and shMPP8_3) were tested for MPP8 depletion (left) and ISRE-GFP reporter induction at day 6 compared to the most effective MPP8 shRNA (called shMPP8_1 here). N=3 biologically independent samples (right) that were pooled to measure MPP8 mRNA in technical duplicates (left). Two-tailed unpaired t tests: p=<0.0001 (shMPP8_1), p=0.0428 (shMPP8_2), and p=0.0407 (shMPP8_3). Control vectors (shEmpty, shControl or shZNF37A) did not induce an IFN response. **b** Extended data from Fig. 1f: MPP8 depletion efficiency in 3 additional cell lines. N=3 biologically independent experiments with technical duplicates shown. **c** Samples from Fig. 1j were used here to verify that the JAK/STAT inhibitor was able to block expression of endogenous ISGs (CXCL10) by qRT-PCR (GAPDH normalized), as well as blocking ISRE-GFP reporter induction. N=2 biologically independent samples. **d** IFN bioassay: supernatants from shRNA-treated 293s were added to ISRE-GFP reporter 293 cells and GFP expression measured 24 hours later. N=4 biologically independent samples. P=0.0817 (not significant, two-tailed paired t test). **e** 293 cells containing an IFN-beta destabilized GFP reporter used in[1] were shRNA-treated and GFP measured 6 days later. N=3 biologically independent samples. P= 0.0037 (two-tailed paired t test). f 293 cells treated with shRNAs in the presence of the JAK/STAT inhibitor Ruxolitinib or DMSO (added from day 1) were harvested for qRT-PCR at day 6. N=3 biologically independent samples with technical duplicates shown. Expression of CXCL10 was normalized to GAPDH. P=0.0454 (two-tailed paired t test). Data presented in this figure show mean values +/- SD, except S1a: left and S1c, which show mean values +/- SEM.

Supplementary Figure 2 MPP8 regulates KZNFs through direct binding.

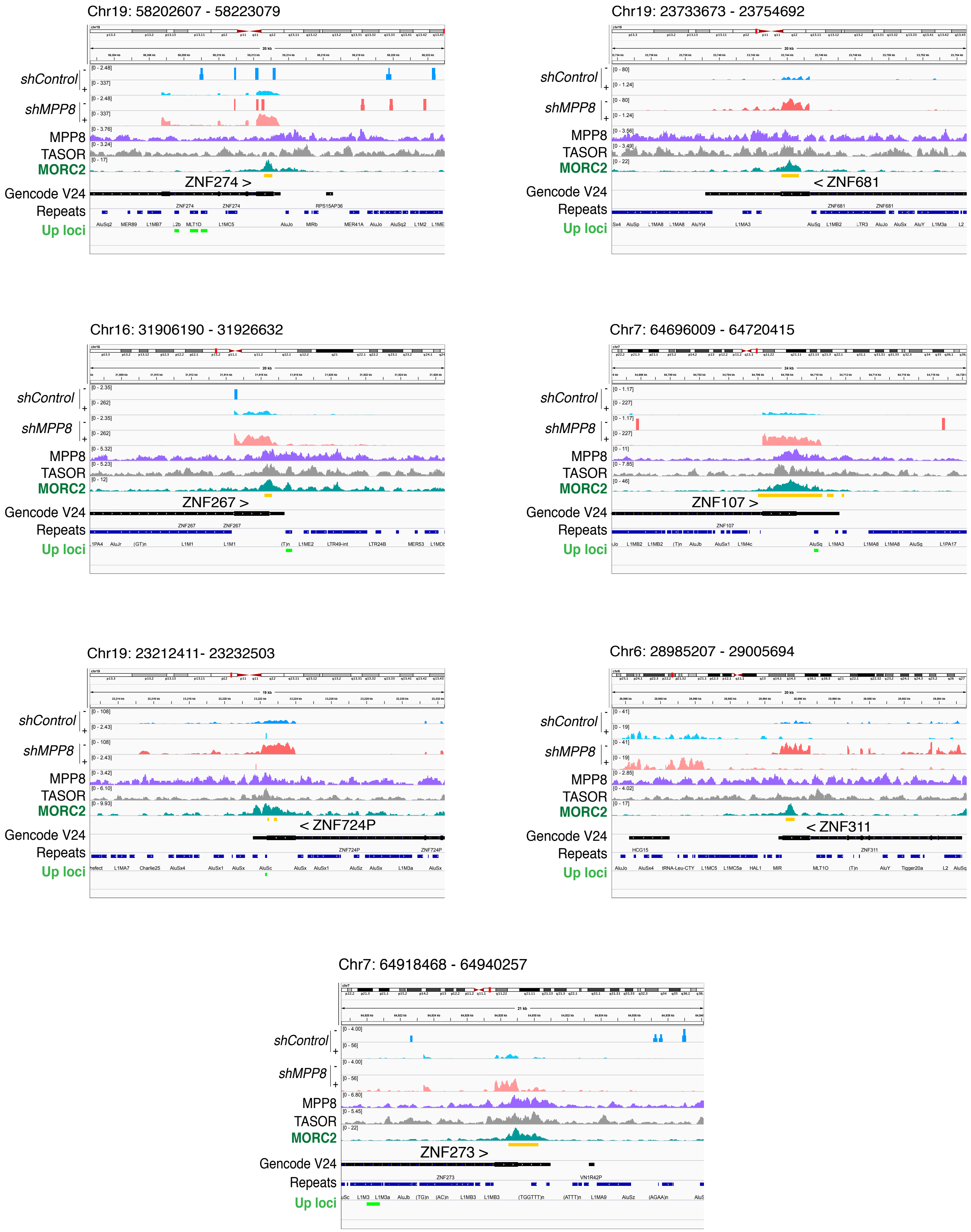

Supplementary Figure 2 MPP8 regulates KZNFs through direct binding.
10 out of 16 KZNFs that are upregulated in MPP8-depleted human primary fibroblasts (HFFs, see Fig. 2b) were identified to be bound by MORC2 using publicly available ChIP data from[2] (based in the leukemia cell line K562), and some examples are shown here. BigWig tracks (see methods) are displayed from total RNA-sequencing data from this study (HFFs, n=3 biologically independent experiments), with normalized scales between *shControl* and *shMPP8*. Underneath, HUSH binding profiles are displayed using public ChIP-seq data from K562 cells[2]. MORC2 ChIP-seq peaks were called over total input samples using MACS2. The MORC2 peaks were used to evaluate binding to KZNF genes but fold enrichment over input tracks for MPP8 and TASOR is also displayed.

Supplementary Figure 3 Functions of genes directly and indirectly regulated by the HUSH complex

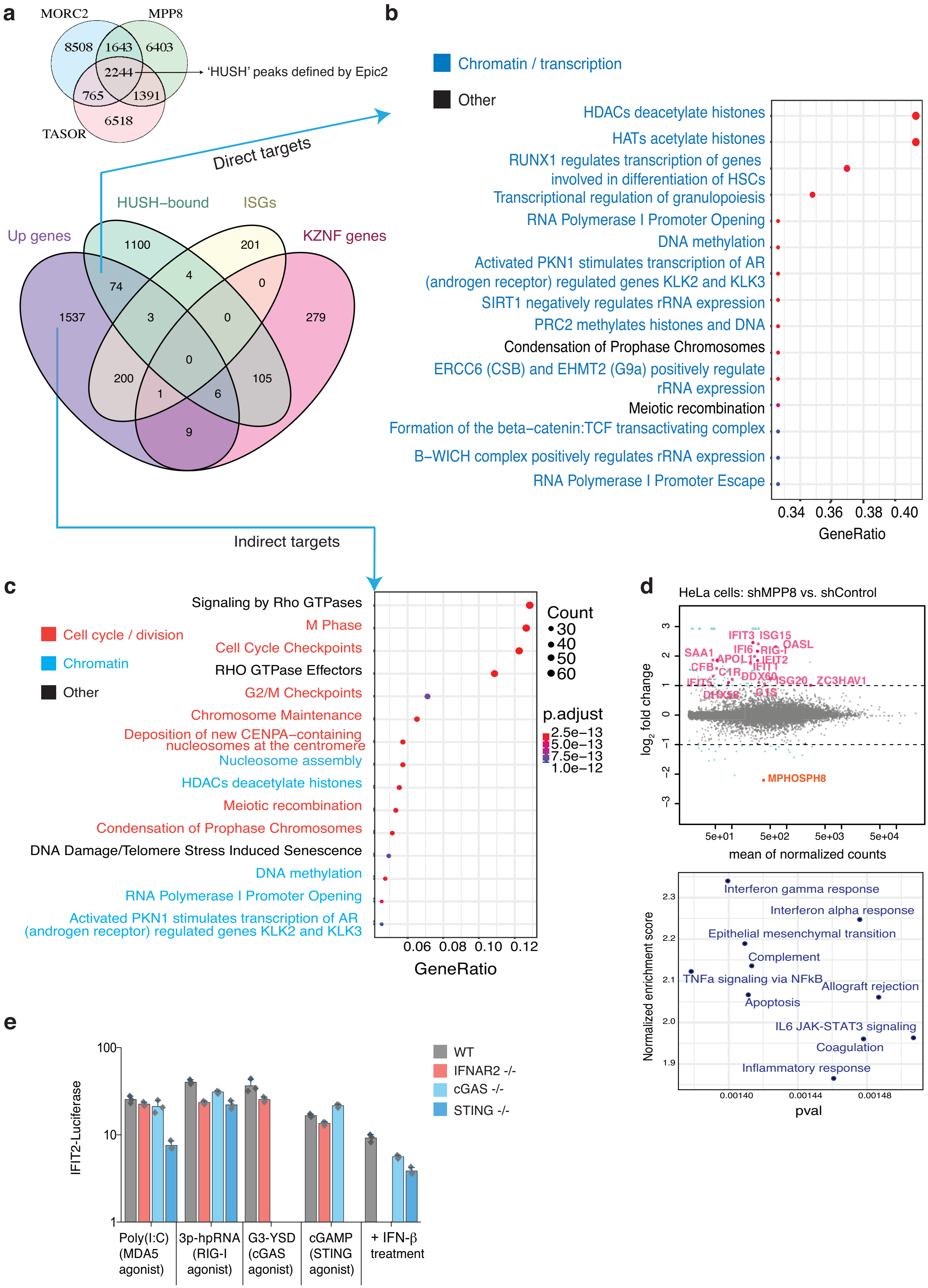

Supplementary Figure 3 Functions of genes directly and indirectly regulated by the HUSH complex.
a Top: Venn-Diagram depicting the number of HUSH complex binding peaks that were uncovered using the overlap of epic2-mapped peaks for MORC2, TASOR and MPP8, using data from[2]. Bottom: Venn-Diagram annotating genes upregulated in *shMPP8* samples compared to *shControls*, using total RNA-seq data from HFFs (N=6 biologically independent experiments) and including HUSH-binding data. **b** REACTOME pathway enrichment analysis of upregulated genes overlapping HUSH peaks which are neither KZFPs nor ISGs (74), where p-values are calculated based on the hypergeometric model and corrected for multiple testing with the FDR method using ReactomePA. **c** REACTOME pathway enrichment analysis of upregulated genes not overlapped by HUSH peaks and which are not annotated as KZFPs nor ISGs (1537), with p-adjusted values calculated as in **b**. **d** HeLa cells were transduced with *shMPP8* or *shControl* vectors and RNA harvested 3 days post *shRNAs* for total RNA-sequencing, confirming that genes involved in immunity are upregulated in an independent cell line. N=3 biologically independent experiments. Upper plot: MA plot showing differentially-expressed genes (log2 fold change > 1 and a p-adjusted value <0.05). Example upregulated ISGs are highlighted as well as MPP8, which is downregulated. Lower plot: Gene set enrichment analyses of upregulated genes in *shMPP8* samples. **e** THP-1 control (WT) or the stated knockout cell lines all harboring an IFIT2-dependent secreted Lucia *luciferase* reporter were treated with the stated agonists and luciferase measurements scored 24 hours later. Background *luciferase* values in non-transfected controls were subtracted from all results. N=3 biologically independent samples. Data are presented as the mean +/- SD.

Supplementary Figure 4 MPP8-depletion results in overexpression of LINE-1s and LTRs

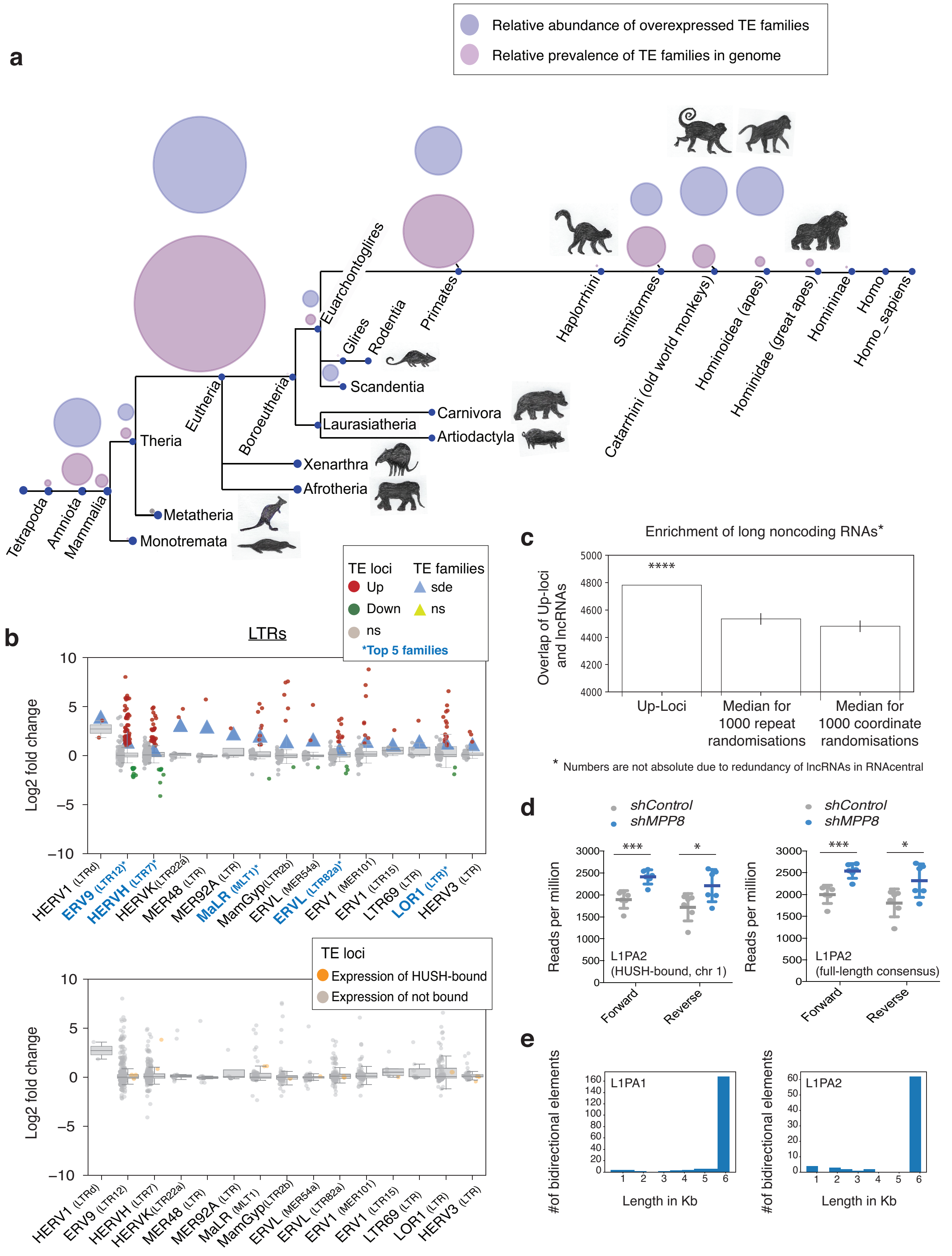

Supplementary Figure 4 MPP8-depletion results in overexpression of LINE-1s and LTRs.
a Complete bubble plot, part of which was shown in Fig. 4c. Relative prevalence of all TE families in the genome by clade (purple) and relative abundance of overexpressed TE families in *shMPP8* samples compared to controls by clade (blue) using RNA-seq data from Fig. 2 and TEtranscripts software[3]. N=6 biologically independent experiments. Animal images drawn by lab members. **b** Upper panel: TE loci up or downregulated in MPP8-depleted cells (log2 fold change >1, and p adjusted values <0.05 after Benjamini-Hochberg multiple testing correction of DESeq2-calculated p-values). See datasets 2 and 3 for exact p values. Results are shown for loci within the stated LTR families, which were the most significantly affected. Differential expression at the family level is also displayed (results from using the software TEtranscripts[3]). Ns = expression not significantly changed. Sde = significantly differentially expressed (p adjusted value <0.05). LTR family names with the highest number of upregulated LTR loci are highlighted in blue. Lower panel: Strip plot showing HUSH-bound (orange) vs. not known to be bound (grey) LTR loci with their relative expression in *shMPP8* samples compared to control. Boxes represent 1st and 3rd quartile, where the central line corresponds to the median; whiskers are 1.5 of the interquartile range. N=6 biologically independent experiments. **c** The overlap of upregulated TE loci, out of 7432 total, and long non-coding RNAs was compared to 1000 randomizations of the same number of random TEs or of random coordinates with the same size and chromosomal distribution as Up-TEs, where a one-sided p value of 0.000999 was calculated as (1+ no. of randomizations with a median greater than the observed value)/1,000 randomizations) . LncRNAs were taken from RNAcentral. **d** The number of RNA-seq reads mapping forward or reverse strands of L1PA2 using a HUSH-bound L1PA2 in the human genome (chr1: 207733982-207734498, left) or using the L1PA2 consensus sequence (right) were plotted for all samples. The L1PA2 consensus sequence was stitched together from its separated parts available on RepBase (L1P1_5end+L1P1_orf2+L1PA2_3end). Two-tailed unpaired t test values: p=0.0007 (L1PA2 chr1 forward) and p=0.0307 (L1PA2 chr1 reverse), p=0.0006 (L1PA2 consensus forward) and p=0.0307 (L1PA2 consensus reverse). N=6 biologically independent experiments. Data show the mean +/- SD. **e** Coordinates of bidirectional LINE-1s were plotted by length in kilobases. Median: 6.029 Kb (L1PA1) and 6.021 Kb (L1PA2).

Supplementary Figure 5 MPP8 regulates young bidirectional LINE-1s and conserved LINE-1s.

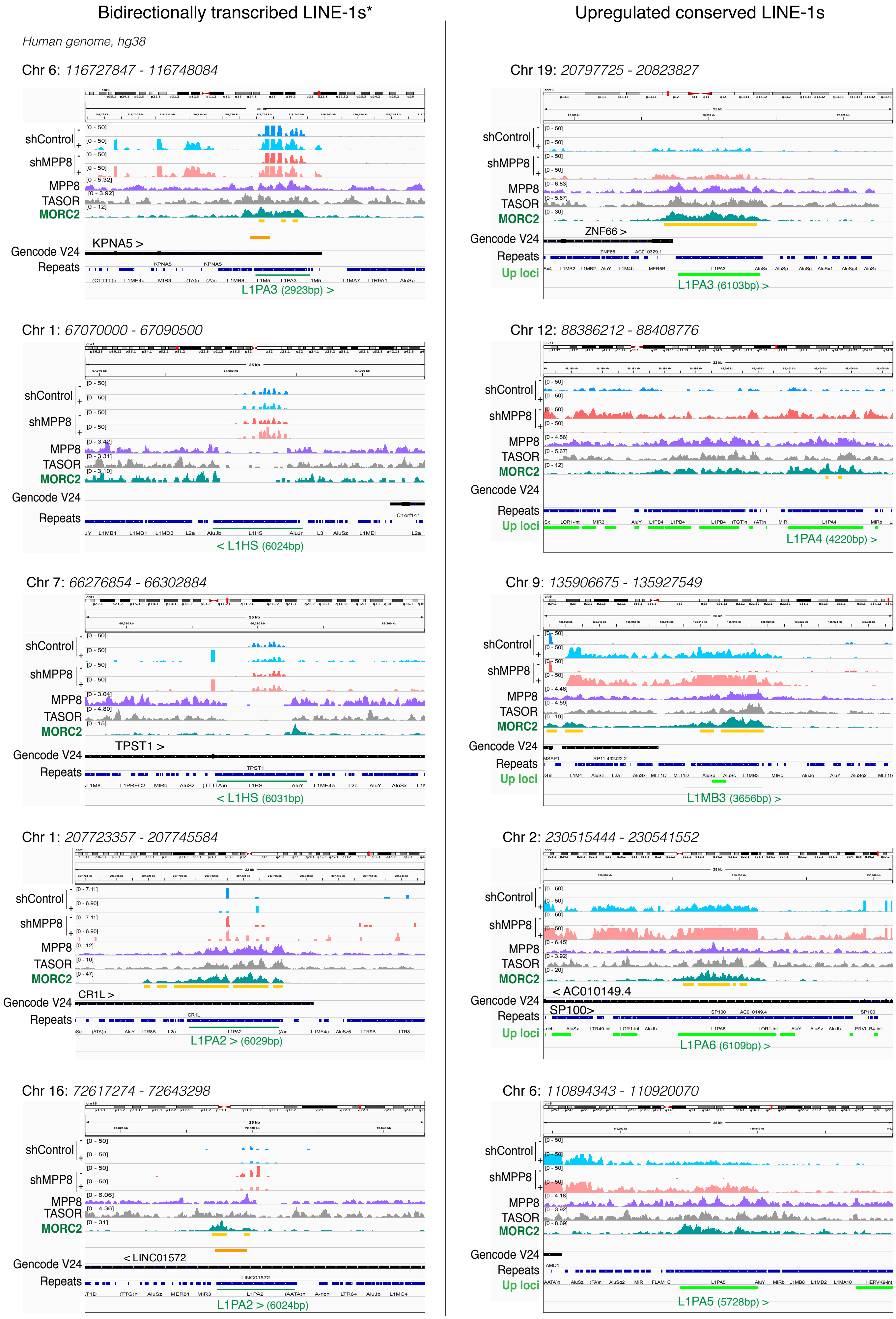

** Note that upregulation of bidirectional L1HS/L1PA2 in the shMPP8 is mainly only detectable when mapping reads to the L1HS/L1PA2 consensus while the above screenshots show evidence for bidirectional expression of loci (in all samples) using only uniquely-mapping reads.*

Supplementary Figure 5 MPP8 regulates young bidirectional LINE-1s and conserved LINE-1s.
BigWig tracks of stranded RNA-seq data (HFFs) mapped to the genome (hg38) are shown for example loci (scales are normalized between *shControl* and *shMPP8* for the same strand). Additionally, ChIP-seq tracks are shown using publicly-available ChIP-seq data (for MPP8, TASOR and MORC2) from K562 cells[2]: MORC2 peaks were called over total input samples using MACS2. On the left are examples of LINE-1 elements that were scored as bidirectionally expressed (mainly L1PA1 and L1PA2). Note that these young LINE-1 elements are already expressed in control cells. Since we are mapping unique reads only to avoid false positives, it is difficult to map a potential increase in L1PA1 expression at the loci level due to mappability issues of young LINE-1s but an overall increase in L1PA1 expression can be mapped by mapping reads to the L1PA1 consensus sequence (see Fig. 4g). On the right are examples of LINE-1 loci scored as upregulated in MPP8-depleted cells (mainly LINE-1s conserved in primates). N=6 biologically independent experiments.

Supplementary Figure 6 LINE-1s drive IFN and MPP8-depletion induces DNA damage.

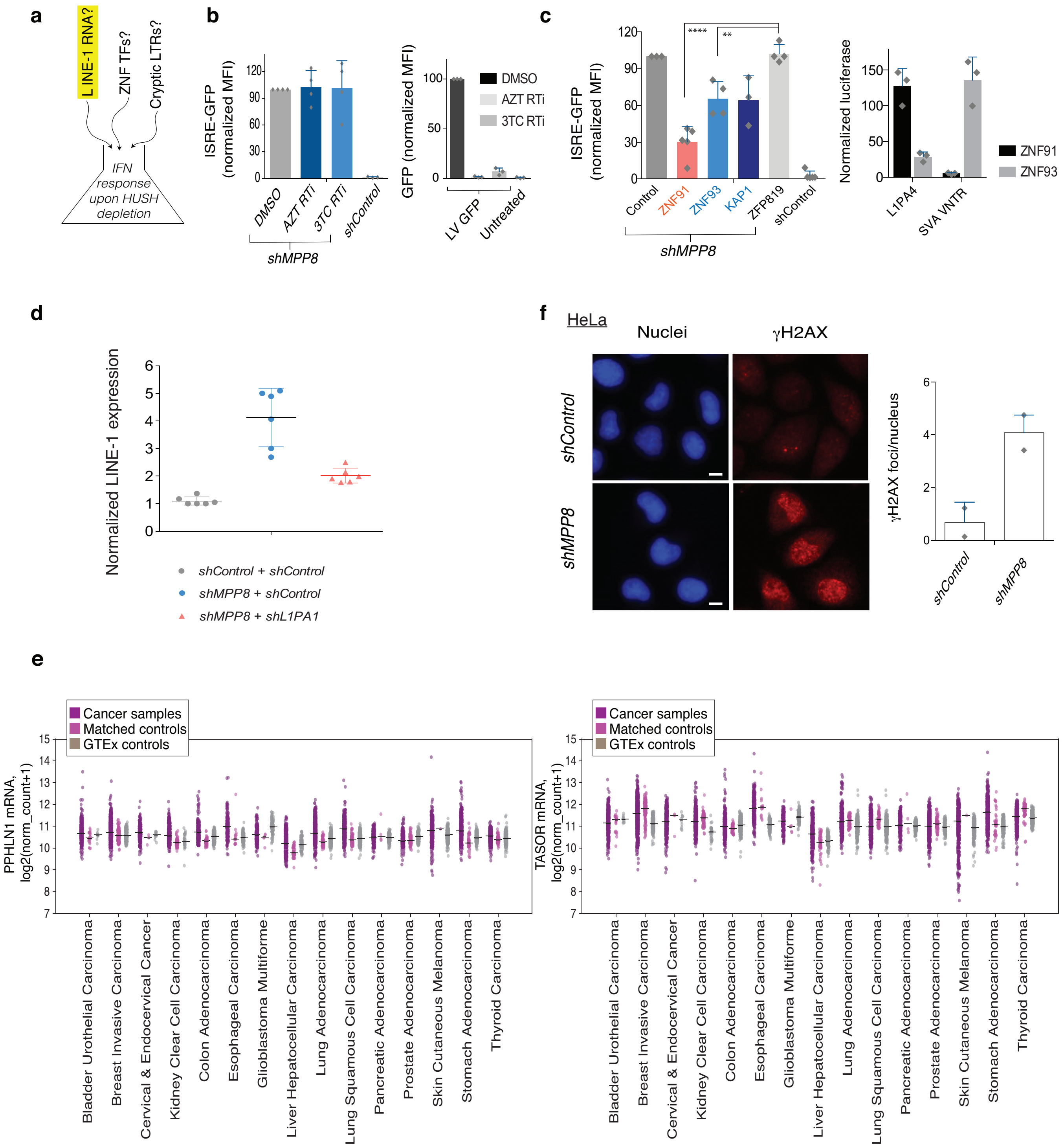

Supplementary Figure 6 LINE-1s drive IFN and MPP8-depletion induces DNA damage.
a While there may be multiple mechanisms contributing to the ISG response in HUSH-depleted cells, we opted to focus on the possible role of LINE-1 elements and related Alu and SVA elements. **b** Left: 293 reporter cells were treated with *shRNAs* in the presence of the stated reverse transcriptase inhibitors (RTis) or DMSO as a control (added from day 1) for the duration of the experiment. GFP was measured by FACS on day 6 and the mean fluorescent intensity (MFI) was set at 100% of the DMSO control and the other samples normalized to this. N=4 biologically independent experiments. Right: In parallel, the same doses of inhibitors were used to block transduction of a PGK-GFP lentivector in 293T cells. N=3 biologically independent experiments. **c** Left: 293 reporter cells were treated with *shRNAs* and transfected the next day with cognate KZNFs/KAP1 or ZFP819 as a control. GFP MFI in control samples (transfection reagent control) were set at 100% and other samples normalized to this. N=5 biologically independent experiments with all treatments each performed in at least 3 experiments. Two-tailed unpaired t test values: p=<0.0001 (ZNF91) and p=0.0070 (ZNF93). Right: In parallel, the stated KZNFs were tested functionally by co-transfecting them with luciferase reporter plasmids containing their binding sites and a renilla control plasmid as used before[4]. N=3 biologically independent samples. **d** 293 reporter cells were co-transduced with the indicated vectors and endogenous expression of LINE-1s was measured using L1PA2-3 primers (see Supplementary Table 2). N=2 biologically independent experiments with technical triplicates shown. **e** Using The Cancer Genome Atlas (TCGA) data, we selected cancers for which there were matched control samples as well as GTEx expression data from the same tissue. *PPHLN1* and *TASOR* expression levels are scored here and are not significantly changed in expression in cancers. See Fig. 6a for *MPP8*. **f** HeLa cells were treated with *shRNAs* before yH2AX staining. N=2 biologically independent samples. Left: Example yH2AX staining images. Right: summary data of yH2AX foci / nucleus. All data show the mean +/- SD, except for S6f, where the mean +/- SEM is shown.

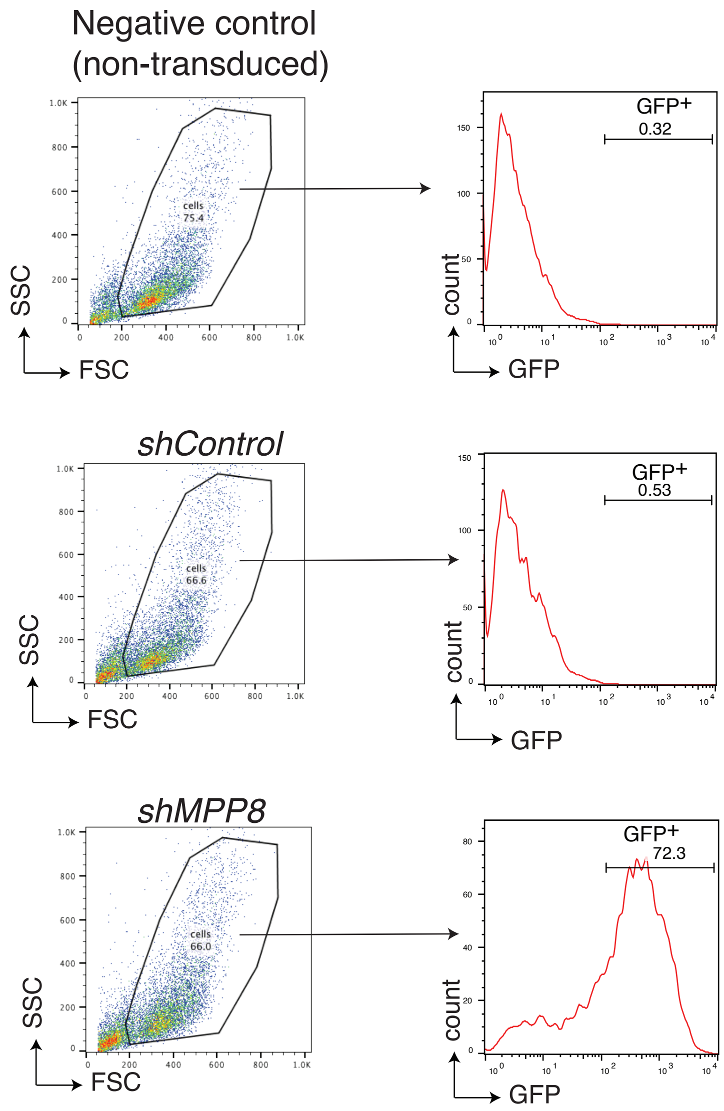

Supplementary Figure 7 Gating strategy used for flow cytometry analyses.
Cells were selected (side scatter (SSC) vs. forward scatter (FSC)) and the level of GFP expression analysed within that population. Non-transduced cells were used as negative controls to define the gate of GFP-positive cells (GFP+) in test samples. When histograms were overlayed from different samples, they were normalized to mode (using Flowjo software) as stated on the y axis.

Supplementary Table 1: shRNA and sgRNA sequences

shRNA sequences

**Hairpin name**	**Hairpin target**	**Designed on**	**No. of matches**	**% L1 matches**	**L1 subfamily with most hits**
shControl	GTTATAGGCTCGCAAAAGG	non-targeting shRNA	0	0	NA
shTASOR	GAGGAAGCTTGAGGATCTA	TASOR CDS (exon 14)	1	0	NA
shPPHLN1	AGCTAACCACTCGCTCTAA	PERIPHILIN CDS (exon 9)	1	0	NA
shMPP8	ACCAGAGACGAAACGGATA	MPP8 CDS (exon 9)	1	0	NA
shMPP8_2	CCGGCAAGCTGTAGTTCTGAATGAT	MPP8 CDS (exon 12), shRNA TRCN0000117925	1	0	NA
shMPP8_3	CCGGGCTAGAGAACAAGAACGCTTT	MPP8 CDS (exon 3), shRNA TRCN0000117926	1	0	NA
shL1PA1_5'UTR	CACCGAAAACCCATCTGTA	L1HS 5'UTR (RepBase)	584	100	(288 copies) and L1PA2 (271 copies)
shL1PA4_ORF2	TGGGCAAGGACTTCATATC	L1PA4 ORF2 on chr11, MPP8-bound using ChIP from: *Liu et a*	136	100	L1PA4 (47 copies)*
shL1_c	ACCCGAATACTGCGCTTTT	L1PA2 5'UTR on chr1, MPP8-bound using ChIP from: *Liu et al.*	678	100	L1PA3 (370 copies)
shL1_d	AGCAAATGCTGAGGGAATT	L1PA10 ORF1 on chr2, MPP8-bound using ChIP from: *Liu et a*	408	100	L1PA10 (135 copies)
shL1_e	GCATAACTGGCTAGCCATA	L1PA15 ORF2 on chr19, MPP8-bound using ChIP from: *Liu et*	31	100	L1PA13 (10 copies)
shL1_f	GCAACATAGATGGAGCTGA	L1PA15 ORF2 on chr19, MPP8-bound using ChIP from: *Liu et*	8	100	L1PA15 (3 copies)
shAlu	CCTGTAATCCCAGCACTTT	Alu (RepBase)	9600	100% Alu	NA
shMOV10	GGGTCAGATATCAGCAAAC	MOV10 CDS (exon 2)	1	0	NA

NA = not applicable

CDS = coding sequence

* this hairpin recognizes 136 LINE-1 loci in total from subfamilies L1PA1 to L1PA5.

sgRNA sequences

Human RIG-I				
**G1**	**score 82**	**negative strand**	**target seq**	**TTGCAGGCTGCGTCGCTGCT**
			**target seq with (**	**GTGCAGGCTGCGTCGCTGCT**
			**Fwd primer**	**CACCGTGCAGGCTGCGTCGCTGCT**
			**Rvs primer**	**AAACAGCAGCGACGCAGCCTGCAC**
G2	score 89	positive strand	target seq	GGATTATATCCGGAAGACCC
			target seq with G	
			Fwd primer	CACCGGATTATATCCGGAAGACCC
			Rvs primer	AAACGGGTCTTCCGGATATAATCC
Human MDA5				
G3	score 92	negative strand	target seq	ATAGCGGAAATTCTCGTCTG
			target seq with (	GTAGCGGAAATTCTCGTCTG
			Fwd primer	CACCGTAGCGGAAATTCTCGTCTG
			Rvs primer	AAACCAGACGAGAATTTCCGCTAC
**G4**	**score 93**	**positive strand**	**target seq**	**TGGTTGGACTCGGGAATTCG**
			**target seq with (**	**GGGTTGGACTCGGGAATTCG**
			**Fwd primer**	**CACCGGGTTGGACTCGGGAATTCG**
			**Rvs primer**	**AAACCGAATTCCCGAGTCCAACCC**

Clones made from sgRNAs in bold are shown in Figure 3

Supplementary Table 2: Primers

qRT-PCR

Primer name	Forward	Reverse
MPHOSPH8 (MPP8)	*TGCCTGTATCTGCCCAAAC*	*CCTTGTCAGAATCATGCCTTTTC*
PPHLN1 (Periphilin)	*GGTTCCAGTGTCAGTAGCAG*	*TCATTCTGCCGTTTGTAGGAG*
FAM208A (TASOR)	*TGAAGACATTGCAGGTTTCATTC*	*CATCCAGGCTATCAACACCAG*
B2M	*TGCTCGCGCTACTCTCTCTTT*	*TCTGCTGGATGACGTGAGTAAAC*
GAPDH	*GGGAAACTGTGGCGTGAT*	*GGAGGAGTGGGTGTCGCTGTT*
IFIT1	*CCTGAAAGGCCAGAATGAGG*	*TCCACCTTGTCCAGGTAAGT*
IFIT2	*ACTATGCCTGGGTCTACTATCA*	*TCAAGCTCTGGACTCTCAATTC*
CCL5	*CCCAGCAGTCGTCTTTGTCA*	*TCCCGAACCCATTTCTTCTCT*
CXCL10	*GTGGCATTCAAGGAGTACCTC*	*GCCTTCGATTCTGGATTCAGA*
MXA	*ATCCTGGGATTTTGGGGCTT*	*CCGCTTGTCGCTGGTGTCG*

qRT-PCR primers against human LINE-1s

Name	*Forward*	*Reverse*	*No. of constrained exact hits**	*% hits to LINE-1s*
L1PA2-3	*GGAAATCATCATTCTCAGTAAAC*	*CACAGTCCCCAGAGTGTGATAT*	*1373*	*100*
L1PA4	*TCACCAATATCCGCTGTTCTG*	*GTCTGTTGGAGTTTACTGGAGG*	*52*	*100*

* Blast hits with 100% identity and full coverage, where the forward and reverse primer hits are located within 200 bps of each other on opposite strands

Breakdown of repeat primer hits to repeat subfamilies

L1PA2-3 primers

No. of hits	Repeat
1219	*L1PA2*
145	*L1PA3*
9	*L1PA1*
1	*L1P1*

L1PA4 primers

No. of hits	Repeat
43	*L1PA4*
5	*L1P1*
2	*L1PA5*
1	*L1PA6*
1	*L1PA3*

Supplementary References

1. Hertzog, J. *et al.* Infection with a Brazilian isolate of Zika virus generates RIG-I stimulatory RNA and the viral NS5 protein blocks type I IFN induction and signaling. *Eur J Immunol* (2018).
2. Liu, N. *et al.* Selective silencing of euchromatic L1s revealed by genome-wide screens for L1 regulators. *Nature* **553**, 228-232 (2018).
3. Jin, Y., Tam, O.H., Paniagua, E. & Hammell, M. TEtranscripts: a package for including transposable elements in differential expression analysis of RNA-seq datasets. *Bioinformatics* **31**, 3593-9 (2015).
4. Jacobs, F.M. *et al.* An evolutionary arms race between KRAB zinc-finger genes ZNF91/93 and SVA/L1 retrotransposons. *Nature* **516**, 242-5 (2014).
