# Supplementary material for: The HUSH complex is a gatekeeper of type I interferon through epigenetic regulation of LINE-1s
Source: Nat Commun. 2020 Nov 3;11:5387. doi: 10.1038/s41467-020-19170-5 (PMC7609715; doi:10.1038/s41467-020-19170-5)
Supplement: Supplementary file 3 — Reporting Summary [file 41467_2020_19170_MOESM3_ESM.pdf]

nature research

Corresponding author(s): Helen M Rowe

Last updated by author(s): Sep 8, 2020

Reporting Summary

Nature Research wishes to improve the reproducibility of the work that we publish. This form provides structure for consistency and transparency in reporting. For further information on Nature Research policies, see our Editorial Policies and the Editorial Policy Checklist.

Statistics

For all statistical analyses, confirm that the following items are present in the figure legend, table legend, main text, or Methods section.

n/a	Confirmed	
☐	☒	The exact sample size (*n*) for each experimental group/condition, given as a discrete number and unit of measurement
☐	☒	A statement on whether measurements were taken from distinct samples or whether the same sample was measured repeatedly
☐	☒	The statistical test(s) used AND whether they are one- or two-sided<br>*Only common tests should be described solely by name; describe more complex techniques in the Methods section.*
☒	☐	A description of all covariates tested
☐	☒	A description of any assumptions or corrections, such as tests of normality and adjustment for multiple comparisons
☐	☒	A full description of the statistical parameters including central tendency (e.g. means) or other basic estimates (e.g. regression coefficient) AND variation (e.g. standard deviation) or associated estimates of uncertainty (e.g. confidence intervals)
☐	☒	For null hypothesis testing, the test statistic (e.g. *F*, *t*, *r*) with confidence intervals, effect sizes, degrees of freedom and *P* value noted<br>*Give P values as exact values whenever suitable.*
☒	☐	For Bayesian analysis, information on the choice of priors and Markov chain Monte Carlo settings
☒	☐	For hierarchical and complex designs, identification of the appropriate level for tests and full reporting of outcomes
☒	☐	Estimates of effect sizes (e.g. Cohen's *d*, Pearson's *r*), indicating how they were calculated

Our web collection on statistics for biologists contains articles on many of the points above.

Software and code

Policy information about availability of computer code

Data collection

BD CellQuest or BD FACSDiva for flow cytometry data acquisition.
Samples were sequenced on a NextSeq 500 instrument (Illumina Cambridge, Chesterford, UK).
Illumina' s bcl2fastq Conversion Software v2.19 was used to demultiplex data.

Data analysis

Flowjo 10.3.0 for flow cytometry analyses,
ImageJ 1.51s for immunofluorescence analyses,
Sequencing quality check - FastQC v.0.11.8,
Read adaptor trimming - TrimGalore v0.4.1,
Read alignment - Tophat 2.1.0 and bowtie 2.4.1,
Read counting per feature - HTSeq-Count 0.11.2,
Differential expression analysis - DESeq2_1.22.1 from R Bioconductor,
Gene set enrichment analysis - fgsea_1.8.0, ReactomePA_1.30.0 and clusterProfiler v3.14.3 R Bioconductor,
Data visualization - Integrative Genomics Viewer from the Broad Institute 2.4.14 , VennDiagram_1.6.20 for R, matplotlib 2.2.3 for python 2.7, and ete3 3.1.1 for python 3. Adobe Illustrator CC 2018,
Repeat quantification - TEtranscript 2.2.0,
TCGA analysis - xenaPython 1.0.10,
Other code has been deposited in https://github.com/regmdr/HUSH_analysis and is detailed in the methods.

For manuscripts utilizing custom algorithms or software that are central to the research but not yet described in published literature, software must be made available to editors and reviewers. We strongly encourage code deposition in a community repository (e.g. GitHub). See the Nature Research guidelines for submitting code & software for further information.

Data

Policy information about availability of data

All manuscripts must include a data availability statement. This statement should provide the following information, where applicable:

- Accession codes, unique identifiers, or web links for publicly available datasets
- A list of figures that have associated raw data
- A description of any restrictions on data availability

Total RNA-sequencing data are available on the NCBI Gene Expression Omnibus database (accession number, GSE135765 [https://www.ncbi.nlm.nih.gov/geo/query/ acc.cgi?acc=GSE135765]) and other data are included in this article and its supplementary information files. Accession numbers for the publicly-available data are: GSE95374 for ChIP-sequencing data on the HUSH complex [https://www.ncbi.nlm.nih.gov/geo/query/acc.cgi?acc=GSE95374], and GSE119999 for RNA-sequencing data on cells expressing engineered LINE-1s [https://www.ncbi.nlm.nih.gov/geo/query/acc.cgi?acc=GSE119999]. Source data are available for Fig. 1b, Fig. 3a, Fig. 3d, Fig. 3e, Fig. 4b, Fig. 5e and datasets for RNA-seq data as a source data file.

Field-specific reporting

Please select the one below that is the best fit for your research. If you are not sure, read the appropriate sections before making your selection.

☒ Life sciences ☐ Behavioural & social sciences ☐ Ecological, evolutionary & environmental sciences

For a reference copy of the document with all sections, see nature.com/documents/nr-reporting-summary-flat.pdf

Life sciences study design

All studies must disclose on these points even when the disclosure is negative.

Sample size	Sample sizes were chosen to reach 3 independent biological replicates to reach biological robustness and are stated in figure legends.
Data exclusions	Data were not excluded from the analyses.
Replication	Experiments were reproduced 3 times and appropriate positive and negative controls were used.
Randomization	Allocation of all samples into experimental groups was random and groups were treated the same.
Blinding	The investigators who carried out the experiments were not blinded to the groups and we did not consider blinding necessary because all groups were treated the same way.

Reporting for specific materials, systems and methods

We require information from authors about some types of materials, experimental systems and methods used in many studies. Here, indicate whether each material, system or method listed is relevant to your study. If you are not sure if a list item applies to your research, read the appropriate section before selecting a response.

Materials & experimental systems

n/a	Involved in the study	
☐	☒	Antibodies
☐	☒	Eukaryotic cell lines
☒	☐	Palaeontology and archaeology
☒	☐	Animals and other organisms
☒	☐	Human research participants
☒	☐	Clinical data
☒	☐	Dual use research of concern

Methods

n/a	Involved in the study	
☒	☐	ChIP-seq
☐	☒	Flow cytometry
☒	☐	MRI-based neuroimaging

Antibodies

Antibodies used	anti-MPP8, #16796-1-AP, (polyclonal) Proteintech, at 1:1000; anti-TASOR, HPA017142, (polyclonal) Atlas Antibodies, at 1:1000; anti-PPHLN1, HPA038902 (polyclonal), Atlas Antibodies, at 1:1000; anti-MDA5, D74E4, #5321, Cell Signaling Technology, at 1:1000; anti-RIG-I, D14G6, #3743, Cell Signaling Technology, at 1:250; anti-MAVS, #3993, (polyclonal) Cell Signaling Technology, at 1:1000; anti-PCNA, NA03, (PC10) Calbiochem, at 1:5000; anti-L1 ORF1p, MABC1152, (4H1) Millipore, at 1:250; anti-alpha-Tubulin, T6074, (B-5-1-2) Sigma Aldrich, at 1:2000; anti-STING (D2P2F), #13647, Cell Signaling Technology, 1:2000; anti-cGAS (D1D3G), #15102, Cell Signaling Technology, 1:2000; anti-IFIT1 (D2X9Z), #14769, Cell Signaling Technology, 1:2000; anti-ISG15 (A-5), sc-166712, Santa Cruz Biotechnology, 1:1000; anti-Actin-HRP (13E5), #5125, Cell Signaling Technology, 1:5000; anti-β-Tubulin-HRP (9F3), #5346, Cell Signaling Technology, 1:5000; anti-dsRNA J2 Ab, 10010200, batch J2-1611 (SCICONS), 0.2ug/test; anti-dsRNA K1 Ab, 10020200, batch

	K1-1601 (SCICONS), 0.2ug/test. Alexa Fluor 488 goat anti-mouse IgG (H+L) secondary Ab, A-11001, Thermofisher, at 1:2000. anti-gH2AX, 613402 (clone 2F3), BioLegend, at 1:400; Alexa Fluor Plus 594 goat anti-mouse IgG (H+L) secondary Ab, # A32742, Invitrogen, at 1:400.
Validation	Antibodies were validated to react with the human versions of the proteins by the manufacturer (anti-MPP8, anti-MDA5, anti-RIG-I, anti-MAVS, anti-PCNA, anti-L1 ORF1p, anti-Tubulin were validated for Western blot or anti-FAM208a and anti-PPHLN1 were validated for Immunofluorescence). Antibodies were further validated to detect proteins of the expected band sizes by Western blot as shown in Figures 1b, 3a and 5e. The dsRNA Abs were validated in intracellular staining assays as shown in Figure 3f, using transfected dsRNA. The gH2AX Ab was verified before: Mlcochova et al., EMBO J, 2018. https://doi.org/10.15252/embj.201796880.

Eukaryotic cell lines

Policy information about cell lines

Cell line source(s)	HEK293 cells were from ATCC and CRISPR/Cas9 RIG-I or MDA5 knockout derivatives were generated by Annemarthe van der Veen. HEK293 cells with integrated ISRE-GFP or IFNbeta-GFP reporters were a gift from Jan Rehwinkel, Oxford. HeLa cells were obtained from Caetano Reis e Sousa (originally from ATCC). Commercial THP-1 cell lines containing an integrated Lucia luciferase IFIT-2 reporter and WT or knockout for cGAS, STING or IFAR were purchased from Invivogen. Human primary foreskin fibroblasts (HFFs) were a kind gift from Matthew Reeves, UCL (originally from ATCC).
Authentication	Morphology test on microscope and growth monitoring from prior experience with working with these cell types. Knockout cell lines were further verified by Western blot and functional assays as detailed in the methods and figures.
Mycoplasma contamination	Regular mycoplasma testing was performed to confirm cell lines were negative.
Commonly misidentified lines (See ICLAC register)	We did not use misidentified cell lines

Flow Cytometry

Plots

Confirm that:

☒ The axis labels state the marker and fluorochrome used (e.g. CD4-FITC).

☒ The axis scales are clearly visible. Include numbers along axes only for bottom left plot of group (a 'group' is an analysis of identical markers).

☐ All plots are contour plots with outliers or pseudocolor plots.

☒ A numerical value for number of cells or percentage (with statistics) is provided.

Methodology

Sample preparation	Cells were trypsinized, resuspended in culture media and washed and resuspended in PBS and acquired on a BD FACSCalibur.
Instrument	Becton Dickinson FACSCalibur flow cytometer and a Becton Dickinson LSRFortessa flow cytometer.
Software	BD CellQuest was used to collect data from the FACSCalibur and BD FACSDiva was used to collect data from the BD LSRFortessa.<br>All data were analysed using FlowJo software.
Cell population abundance	Cell populations were not sorted on a cell sorter
Gating strategy	For all experiments, live cells were gated on by excluding debris, based on the forward and side scatter profiles. A gate of GFP positive cells was made using the negative control sample and copied to all samples.

☒ Tick this box to confirm that a figure exemplifying the gating strategy is provided in the Supplementary Information.
